# Probiotic Efficacy in Aquaculture: The Role of Technospore® (*Bacillus coagulans*) in Improving Nile Tilapia (*Oreochromis niloticus*) Performance and Disease Resistance: a Study on Gut Health, Immunological Response, and Gene Expression

**DOI:** 10.1007/s12602-024-10279-3

**Published:** 2024-05-21

**Authors:** Amira A. Omar, Mohamed S. Gado, Hamada E. Kandel, Foad A. Farrag, Mustafa Shukry

**Affiliations:** 1 Fish Diseases and Management Department, Faculty of Veterinary Medicine, Kafrelsheikh University, Kafrelsheikh, Egypt; 2 Anatomy and Embryology Department, Faculty of Veterinary Medicine, Kafrelsheikh University, Kafrelsheikh, Egypt; 3 Animal Physiology Department, Faculty of Veterinary Medicine, Kafrelsheikh University, Kafrelsheikh, Egypt; 4 Department of Basic Veterinary Sciences, Faculty of Veterinary Medicine, Delta University for Science and Technology, Dakahlia, 7730103 Egypt

**Keywords:** *Oreochromis Niloticus*, *Bacillus Coagulans*, *Aeromonas Hydrophila*, Growth Performance, Immunological Responses

## Abstract

This study evaluated the effects of Technospore® (*Bacillus coagulans*) supplementation on intestinal health, immune response, and *Oreochromis niloticus* (Nile tilapia) growth performance. The experiment divided fish into four groups: a control group fed an unsupplemented diet and three experimental groups receiving diets supplemented with 0.2 g/kg, 0.4 g/kg, and 0.8 g/kg of Technospore®, respectively. Results indicated that Technospore® supplementation significantly enhanced growth rates and feed efficiency in all treated groups, with the most pronounced improvements observed in the group receiving 0.4 g/kg. Furthermore, the study revealed that *B. coagulans* supplementation markedly boosted serum immune responses, as evidenced by increased phagocytic activity, phagocytic index, and lysozyme levels, following a challenge with *Aeromonas hydrophila*. Histological analysis showed improved gut morphology, while gene expression analysis indicated upregulation of immune-related genes, including liver IGF-1, GHR, HSP70, IL-1β, and TNF-α, as well as spleen TNF-α and IL-1β and intestinal C-lysozyme and TNF-α, both before and after the bacterial challenge. These findings suggest that dietary inclusion of Technospore® can significantly improve gut health and immune responses in tilapia, potentially serving as an effective prophylactic alternative to antibiotics in aquaculture.

## Introduction

Over the last seven years, Egyptian aquaculture has grown dramatically and quickly. The country is now leading Africa, ranking sixth globally in aquaculture production and third globally in tilapia production; this has led to a significant boost in Egypt’s economy and food security. By 2025, Egypt intends to increase fish production to three million metric tons. Excellent fish farming techniques, high-quality feed, and improved water use efficiency are some of the main elements influencing the noteworthy rise in aquaculture productivity. Other essential variables include improved feed usage enhancement technologies [1].

The most common fish species in these ponds is *Oreochromis niloticus*, which contributes around 65% of aquaculture production because of the broad escalation and cultivated regions. *Oreochromis niloticus*, Nonetheless, one of the most prominent fish species is the Nile tilapia, which is widely produced globally due to their ease of reproduction, tolerance to a range of diseases and conditions, quick development, and high consumer demand [2].

Living bacteria that are not harmful are called probiotics, and they strengthen immunity, growth, and the balance of gut microbes in animals. In aquaculture, probiotics are being utilized in place of antibiotics. According to several research, probiotics, such as yeast and lactic acid bacteria, are widely used in aquaculture. *Bacillus* species have been identified as a growing trend in the interim [3–7].

Probiotic strains, including living and dead bacteria, may influence the interspecies interactions among all members of the microbial population, thereby promoting gut microbiota homeostasis. According to previous study findings, probiotics can be administered through diet to enhance the host’s overall state of health by affecting immunological response, nutrition metabolism, and organ growth. The host’s immune system can be stimulated by various substances present in bacterial cell walls, enhancing innate and adaptive defenses against pathogenic infections [8–10].

Gram-positive *Bacillus coagulans* is a bacteria that forms spores due to the confluence of lactic acid bacteria and *Bacillus* characteristics; it is utilized as a feed additive in aquaculture. Moreover, *B. coagulans* has been shown to resist bile salts, high temperatures, and acidity while suppressing enteropathogens. *B. coagulans* promotes microbiota regulation, immune system function, and growth while offering protection from illness and conferring advantages to the organism through colonizing animals’ intestines, enhancing intestinal well-being, and exhibiting resistance against infections [11–17].

TechnoSpore® (*Bacillus coagulans* DSM 32016), submitted for authorization by Biochem Zusatzstoffe Handels- und Produktionsges. mbH is evaluated under the European Commission’s Regulation (EC) No 1831/2003 for use as a zootechnical feed additive in piglets, growing Suidae, chickens for fattening, other poultry, and ornamental birds. Based on EFSA’s rigorous assessment protocol, TechnoSpore® is characterized for its safety and efficacy. The active strain, isolated from canned tomatoes and thoroughly tested, shows no cytotoxic effects or acquired antimicrobial resistance, aligning with the qualified presumption of safety (QPS) criteria. The manufacturing process ensures high viability of spores with strict contaminant controls, presenting a stable, homogenous product suitable for animal feeds. TechnoSpore® is deemed safe for target species, consumers, and the environment, without skin or eye irritation risks, though it is considered a potential respiratory sensitizer for handlers. Efficacy studies support its use in improving gut flora stability in specified animal groups at a concentration of 1.9 × 10^9 CFU/kg feed, with compatibility assessed for certain coccidiostats. Thus, TechnoSpore® meets the EFSA’s safety and efficacy standards for the intended uses, offering a promising additive for animal nutrition [18].

In recent years, there has been an increase in disease outbreaks among farmed fish due to the expansion of intensive aquaculture and the decline of the physiochemical qualities of the water. Numerous fish species have had the biological impacts of *B. coagulans* widely researched, but little is known about how they affect Tilapia performance. Consequently, The main objective of this study was to assess the effects of incorporating *B. coagulans* into the diets of tilapia, focusing on enhancements in growth performance, intestinal histomorphometry, immunological responses, and the modulation of key gene expressions related to these aspects.

## Materials and Methods

### Ethical Approval

All fish manipulation processes were executed to comply with the animal use and care standards set forth by the Kafrelsheikh University Ethics Committee in Egypt (KFS-IACUC/139/2023) for scientific purposes.

### Additives Tested

One commercially accessible product was used in this experiment. Safe feed additives comprising 0.5–1 × 10^9^ CFU/kg final feed have been made using commercial products of *B. coagulans* (Technospore®; Biochem Co., Germany).

### Design of the Experiment and the Conditions of Rearing

Utilizing polypropylene bags with enough pure oxygen in them, three hundred healthy *O. niloticus* (33 ± 0.5 g) were purchased from a private hatchery in Al Rayed, tolompate 7, Kafr El-Sheikh Governorate, Egypt, and transported to the wet lab. of Fish Diseases and Management Dept., Fac. Vet. Med., Kafrelsheikh Uni., Egypt. The transported fish were housed in a fish tank for two weeks to allow them to adjust to the laboratory environment. The fish were randomly placed into four identical groups in each of the three repetitions in a 25-fish glass fish tank. Every day, fish were fed thrice at 8:00, 13:00, and 16:00 for eight weeks until they appeared satiated. The appropriate levels of tilapia-specific physiochemical characteristics and fish-rearing circumstances were sustained. During the feeding trial period. The aquarium measures 80 × 50 × 60 cm, with a photoperiod of 12 hours L - > 12 hours D. The water temperature is 26.5 ± 1 °C, pH is 7.2 ± 0.5, dissolved oxygen is six mg/L, and total ammonia as nitrogen is less than 0.3 mg/L. An eight-week feeding trial was added. During this time, the fish were split into four equal groups: The diet of the control group (CNT) was provided without any supplements. The follow-up groups received different amounts of Technospore® supplements: G1, G2, and G3 received 0.2 g/kg, 0.4 g/kg, and 0.8 g/kg of feed Technospore®^,^ respectively.

### Feed Components and Diet Setup

The elements of the designed diet, which are frequently used in tilapia rations, were corn gluten meal, wheat bran, soybean meal, fishmeal, and yellow corn. The diet consisted of 30% crude protein and was intended to be both isonitrogenous and isocaloric. According to recommendations from [19], The purpose of the baseline feed (Table 1) is to meet the basic nutritional needs of tilapia fish. Each prepared diet was made by carefully combining all the ingredients. The diet was then mixed, and the resulting components, feed additive, and water were amalgamated to create an appropriate blend for each diet. Each diet was pelletized using a lab pellet machine with a 2 to 3-mm diameter. The resultant moist pellets were entirely dried at ambient temperature. Before being used, the pellets that had been dehydrated were kept in opaque plastic containers and refrigerated at −4 °C. After a 24-hour preservation period, the probiotic bacteria’s viable count and activity in the ready-to-eat diets were assessed following [20] protocols.

**Table 1:** The basal diet’s formulation and chemical constituents (% dry matter)

Feed Ingredients				
(CNT)	Control	G1	G2	G3
Fish meal (72% CP)	110	110	110	110
Soybean meal (45% CP)	360	360	360	360
Wheat bran	200	200	200	200
Yellow corn	60	60	60	60
Rice bran	200	200	199	198
Technospore3®	0	0.2	0.4	0.8
Fish oil	15	15	15	15
Soybean oil	15	15	15	15
Dicalcium phosphate	10	10	10	10
Vitamins mixture^a^	10	10	10	10
Minerals mixture^b^	10	10	10	10
Carboxymethyl cellulose	10	10	10	10
Total	1000	1000	1000	1000
Chemical analysis				
Dry matter	91.5	91.3	91.8	91.2
Crude protein	31.3	31.3	31.3	31.3
Ether extract	8.1	8.1	8.1	8.1
Total ash	7.33	7.37	7.3	7.31
Crude fiber	6.6	6.68	6.65	6.62
Nitrogen-free extract	46.7	46.4	46.7	46.5
Gross energy (kcal/g)^c^	4.71	4.72	4.74	4.7

^a^Vitamin mixture (per kg of premix): pyridoxine, 2.0 g thiamine, 2.5 g, biotin, 0.3 g, riboflavin, 2.5 g, inositol, 100.0 g, folic acid, 0.75 g, pantothenic acid, 100.0 g, para-aminobenzoic acid, 2.5 g, nicotinic acid, 10.0 g choline, 200.0 g, cyanocobalamine, 0.005 g, cholecalciferol, 500,000 IU, a-tocopherol acetate, 20.1 g, retinol palmitate, 100,000 IU menadione, 2.0 g

^b^Mineral mixture (g/kg of premix): MgCO_4_.7H_2_O, FeC_6_H_5_O_7_.3H_2_O, 25.0, KCl 50.0, NaCl, 60.0, 127.5, CaHPO_4_.2H_2_O, 727.2, CoCl_3_.6H_2_O, 0.477, ZnCO_3_, 5.5, MnCl_2_.4H_2_O, 2.5, CrCl_3_.6H_2_O, 0.128, Cu(OAc)_2_.H_2_O, 0.785, AlCl_3_.6H_2_O, 0.54, CaIO_3_.6H_2_O, 0.295, Na2SeO_3_, 0.03

^c^Gross energy (GE) was calculated from [18] as 4.11, 9.45, and 5.65 kcal/g for carbohydrates, lipids, and protein, respectively

### Growth Measurements and Feed Efficiency

Fish were fed and weighed every two weeks to determine the eaten meals and growth. To evaluate the effectiveness of the fish diet that was consumed, various parameters were considered and determined following [18], including average daily gain (ADG), weight gain (WG), final body weight (FBW), specific growth rate (SGR), feed intake (FI), feed conversion rate (FCR), and survival rate.

### Sampling

Before blood collection, Tetracaine methane-sulfonate, manufactured by Sigma-Aldrich Co. LLC, possessed a sedated concentration of 100 μg ml ^−1^ and was used to sedate the fish. Each aquarium’s nine fish were chosen randomly for each treatment to supply blood samples [21]. Utilizing sterile 2.5 ml syringes, blood samples were extracted from the caudal vein and divided into equal portions. Regarding hematological assessments, the first portion was kept in a heparinized tube. On the other hand, the second part was kept at 4 °C for three hours after being left to coagulate for thirty minutes at room temperature. After centrifuging the clotted samples for 10 minutes at 3000 rpm at 4 °C, serum was extracted and preserved at −20 °C to continue biochemical and immunological examination. Tissue samples from the anterior region of the intestine, hepatopancreas, kidney, and spleen were collected from five randomly chosen fish from each treatment group (control, G1, G2, G3), both before and after exposure to *Aeromonas hydrophila*. For gene expression analysis, parts of the tissues were immediately frozen at −80 °C, while other parts were fixed in a 10% formaldehyde solution for 24 to 48 hours for histopathological examinations.

### Hematological Evaluations of the Blood

The Natt-Herrik solution and a hemocytometer were used to estimate the numbers of leukocytes and erythrocytes after the [22] process. As recommended by [23], the cyanmethemoglobin technique was used to measure the hemoglobin level. Furthermore, the microhematocrit method performed the PCV% estimate and the computation of MCHC, MCH, and MCV [24]. The procedure described in reference [25] was used to create blood smear slides, air dry them, fix them with methanol for three to five minutes, stain them with Giemsa for eight to ten minutes, After giving them a quick wash in water that was distilled, let them air dry at ambient temperature to calculate the differential leukocytic count.

### Biochemical Analysis of Blood

The calorimetric technique was employed to evaluate the protein content of the blood (total protein; albumin); conversely, the globulin level was calculated by deducting the albumin value from the total protein concentration. Moreover, commercial kits (Assay Kit, 384 well, Colorimetric/Fluorometric, ABACM241035) were used to assess the enzymes of liver function; aspartate aminotransferase (AST) and alanine aminotransferase (ALT) following [26] protocol.

### Immunological Parameters

According to the protocol in [27], the lysozyme activity was measured at 450 nm using the USA-based Sigma *Micrococcus lysodeikticus* approach utilizing a microplate ELISA reader. The following was used to assess phagocytic activity (PA) and phagocytic index (PI) [28, 29].

### Challenge with *A. hydrophila*

The experimental infection aimed to ascertain how Technospore® feed supplementation affected the *Oreochromis niloticus*’ ability to fend off *Aeromonas hydrophila* bacterial infection. According to [30], 0.2 ml of fresh *A. hydrophila* culture solution administered intraperitoneally (3 × 10^7^ CFU). It was used for the experimental infection. Daily mortalities have been documented for 15 days after infection.

The concentration of *A. hydrophila* strain per 1 ml and the dosage for the experimental investigations were estimated using the drop plate method [31]. A 24-hour colony culture of the *A. hydrophila* strain was employed for plate counts on TSA after being lifted and suspended in a sterilized saline solution through a 10-fold serial dilution; a whole day was spent incubating the colonies at 28 °C. The dilutions (10^2^ to 10^7 ^CFU) were used only. Every group received an intraperitoneal injection of 0.5 ml per fish for every bacterial dilution. For two weeks following injection, every fish was housed for observation. [32] stated that the fatalities were documented twice a day. The recently deceased fish were relocated for a more thorough investigation.. The LD_50_ (the dosage that kills 50% of the inoculated fish) was determined [33].

### RNA Extraction and Quantitative Real-Time PCR

In compliance with the manufacturer’s instructions, TRIzol reagent was used to extract total RNA using the (Life Technologies, Gaithersburg, MD, USA). The purity and concentration of the RNA extracted were verified using a Nanodrop (UV-Vis spectrophotometer Q5000/Quawell, USA). After that, the MultiScribe kits of RT enzyme (Applied Biosystems,USA) were utilized to synthesize cDNA. The analysis of triplicate real-time PCR was performed on the resultant cDNA using a System of 7500 Real-Time PCR (Foster City, California, USA: Applied Biosystems) and Master Mix for Power SYBR Green PCR (Applied Biosystems, USA). The variation in fold in the mRNA levels of the measured genes was normalized using the expression of GAPDH, a typical housekeeping gene. Different genes’ mRNA expression fold change was evaluated compared to the control. Table 2 contains the gene accession codes and primer sequences. Thus, the 2^-∆∆Ct^ approach was used to equalize the target gene’s critical threshold (Ct) quantities with the gene of housekeeping amounts (Ct) [34].

**Table 2:** The appropriate primer sequences utilized in real-time q-PCR analysis of gene expression

Genes	Forward 5′- > 3′	Reverse 5′- > 3′	Efficiency %	Accession No.
IL-1β	TGCTGAGCACAGAATTCCAG	GCTGTGGAGAAGAACCAAGC	99.2145	KF747686.1
HSP70	GGAGTCCTACGCCTTCAACA	CAGGTAGCACCAGTGGGCAT	96.2051	FJ213839.1
IGF-1	TGCCCAAGACTCAGAAGGAAG	ATCCTGTAGTTCTTGTTTCCTGCAC	94.3268	NM_001279503.1
TNF-α	GAGGCCAATAAAATCATCATCCC	CTTCCCATAGACTCTGAGTAGCG	95.6571	JF957373.1
GHR	CTCACTGACTGGGACCACAC	GGAGGAGAGGTTGTGGAAGC	97.8457	EF052861
C-lysozyme	AGGGAAGCAGCAGCAGTTGTG	CGTCCATGCCGTTAGCCTTGAG	99.5687	XM_003460550.2
GAPDH	TGTTTGTGATGGGCGTGAA	CCTCCACAATGCCGAAGT	96.3657	NM_001082253.1

### Histopathological Examination and Morphometric Analysis

Tissue samples were fixed for 24 to 48 hours in a 10% formaldehyde solution; the samples were dehydrated using ethyl alcohol at progressively higher concentrations (70% to 100% alcohol). Following dehydration, the samples were embedded in paraffin wax after being washed in xylene. Sections measuring 4–5 μm in thickness were stained using hematoxylin and eosin for histopathological and morphometric examination [35]. Two pathologists worked together to score the histopathological lesions. The crypt depth, width, and length of the intestinal villi were measured using image analysis tools from the National Institutes of Health in Bethesda, MD, and the villi length/crypt depth for morphometric analysis. The data was tested using a one-way ANOVA using SPSS version 22, SPSS Inc., Illinois, USA., acquired by randomly selecting ten villi and crypts related to villus from each of the five intestinal cross-sections. A significant threshold of (*p* < 0.05) was agreed upon. The means ± standard error are displayed for every data.

### Statistical Analysis

Kolmogorov–Smirnov test was employed to check the data obtained for normality patterns. The effects of dietary Technospore®, challenge, and their interactions were tested using two-way ANOVA. Once the interaction between the two factors was identified, One-way ANOVA was utilized to investigate each of the four groups for every level of the two factors combined. Tukey-Kramer was employed in the data comparison from each group to that of the control group, which was also used to look at differences between sampling periods and within the same group for each parameter. One-way ANOVA testing evaluated all statistical differences (SPSS version 22, SPSS Inc., Il, USA). In cases of differences between experimental groups, the Duncan post-hoc test was employed. The statistically significant threshold was considered acceptable at (*p* < 0.05). Every data set is shown as means ± standard error.

## Results

The current study investigated the physiological responses of Nile tilapia to Technospore® by ensuring fish growth and immunological, biochemical, hematological, and intestinal health indices.

### Growth Performance and Feed Utilization

In conclusion, in the feeding study, fish groups fed Technospore® had significantly larger final weights, body weight gains, and average daily gains (*p <* 0.05) than the unsupplemented diets given to the group of control (Table 3). Also, compared to those in the control group, feed supplementation with Technospore® might notably improve SGR. Although there were no appreciable differences in FCR values between the supplemented groups (*p* > 0.05), the FCR values in the supplemented groups were substantially less than those recorded in the control group (*p* < 0.05). There existed no notable distinctions between the groups that received supplements (*p* > 0.05), even though the supplemented groups’ feed intake and survival rate were much higher than the control group (*p* < 0.05).

**Table 3:** Effects of dietary supplementation with Technospore® on growth performance of *Oreochromis niloticus*

Parameters	CNT	G1	G2	G3
Initial fish weight(g)	33.5 ± 0.25 ^ab^	33.5 ± 0.50 ^ab^	33.93 ± 0.05 ^a^	33.86 ± 0.15 ^ab^
Final fish weight (g)	50.5 ± 0.50 ^e^	56.23 ± 0.25 ^b^	59.00 ± 1.00 ^a^	59.40 ± 0.40 ^a^
Weight gain (g)	17.25 ± 0.75 ^e^	22.73 ± 0.25 ^b^	25.06 ± 0.95 ^a^	25.53 ± 0.25 ^a^
ADG	0.30 ± 0.01 ^e^	0.40 ± 0.01 ^bc^	0.43 ± 0.02 ^ab^	0.45 ± 0.01 ^a^
FCR	1.53 ± 0.35 ^a^	1.33 ± 0.01 ^c^	1.22 ± 0.02 ^d^	1.20 ± 0.05 ^d^
SGR, %/fish/day	5.08 ± 0.08 ^e^	5.57 ± 0.02 ^bc^	5.67 ± 0.07 ^a^	5.78 ± 0.02 ^a^
Feed intake	26.28 ± 1.87 ^b^	30.58 ± 022 ^a^	29.43 ± 0.56 ^a^	30.64 ± 0.97 ^a^
Survival rate (%)	90.00 ± 2.00 ^b^	100.00 ± 0.00 ^a^	100.00 ± 0.00 ^a^	100.00 ± 0.00 ^a^

The data represent the mean ± SE. A different superscript for the same numbers indicates notable variations (*p* < 0.05) in each row and for (*P* > 0.05), not significant. CNT control group, G1 = (0.2 g/kg Technospore®), G2 = (0.4 g/kg Technospore®), G3 = (0.8 g/kg Technospore®), *SGR* specific growth rate, *FCR* feed conversion ratio, *ADG* Average Daily Gain

### Blood Hematological Differentiations

All treated groups had significantly greater RBCs and HB levels (*p* < 0.05) than the control, with the highest level in G2. However, there were no appreciable variations (*p* > 0.05) in MCV among the supplemented and control groups. PCV was considerably higher in G2 and G3 (*p* < 0.05) than the control group, while the G1 and control groups did not differ significantly. Compared to the control group, MCH and MCHC notably increased in all treated groups (*p* < 0.05). (Table 4). Regarding differential leukocytic count, the findings showed there were no noteworthy distinctions between G1 and the control group, significantly (*p* < 0.05); there was an increase in WBCs and heterophil in the G2 and G3 groups than the control group. All supplemented groups displayed a substantial (*p* < 0.05) drop in monocyte and lymphocyte percentage compared to the control group. The percentages of basophil and eosinophil in treated and control groups remained unchanged (*p* < 0.05), according to Table 5.

**Table 4:** Effect of Technospore® on hematological parameters of *Oreochromis niloticus*

Parameters	CNT	G1	G2	G3
RBCS (×10^6^/mm^3^)	3.28 ± 0.01 ^c^	3.29 ± 0.02 ^c^	3.48 ± 0.06 ^a^	3.41 ± 0.01 ^ab^
HB (g/100 ml)	9.89 ± 0.01 ^d^	10.08 ± 0.06 ^cd^	10.70 ± 0.11 ^a^	10.45 ± 0.14 ^b^
PCV (%)	32.00 ± 0.00 ^b^	32.00 ± 0.00 ^b^	34.00 ± 0.00 ^a^	33.50 ± 0.50 ^a^
MCV	97.56 ± 0.30	97.27 ± 0.59	97.59 ± 1.82	98.09 ± 1.03
MCH	30.16 ± 0.45 ^b^	30.63 ± 0.01 ^a^	30.72 ± 0.24 ^a^	30.61 ± 0.29 ^a^
MCHC	30.92 ± 0.45 ^c^	31.50 ± 0.19 ^ab^	31.48 ± 0.33 ^ab^	31.21 ± 0.30 ^abc^

The data represent the mean ± SE. A different superscript for the same numbers indicates notable variations (*p* < 0.05) in each row, and for (*P* > 0.05), not significant. CNT = (control group), G1 = (0.2 g/kg Technospore®, G2 = (0.4 g/kg Technospore®, G3 = (0.8 g/kg Technospore®, *RBCs* Red Blood Cells, *HB* Hemoglobin, *MCV* Mean corpuscular volume, *MCH* Mean corpuscular hemoglobin, *PCV* Packed Cell Volume, *MCHC* Mean corpuscular hemoglobin concentration

**Table 5:** Effect of Technospore ® on the differential leukocytic count of *Oreochromis niloticus*

Parameters	CNT	G1	G2	G3
WBCs (%)	21.10 ± 0.93 ^c^	21.93 ± 0.89 ^c^	24.46 ± 1.43 ^b^	25.36 ± 0.25 ^ab^
Lymphocyte (%)	16.50 ± 1.50 ^a^	13.00 ± 0.00 ^b^	10.50 ± 0.50 ^c^	11.00 ± 1.00 ^c^
Heterophil (%)	72.50 ± 1.50 ^c^	78.00 ± 0.00 ^b^	79.00 ± 0.00 ^ab^	79.00 ± 1.00 ^ab^
Monocyte (%)	9.00 ± 0.00 ^a^	7.00 ± 0.00 ^d^	8.50 ± 0.00 ^ab^	8.00 ± 0.00 ^bc^
Basophil (%)	1.00 ± 0.00	1.00 ± 0.00	1.00 ± 0.00	1.00 ± 0.00
Eosinophil (%)	1.00 ± 0.00	1.00 ± 0.00	1.00 ± 0.00	1.00 ± 0.00

The data represent the mean ± SE. A different superscript for the same numbers indicates notable variations (*p* < 0.05) in each row, and for (*P* > 0.05), not significant. CNT = (control group), G1 = (0.2 g/kg Technospore®), G2 = (0.4 g/kg Technospore®), G3 = (0.8 g/kg Technospore®), *WBCs*, white blood cell count

### Blood Biochemical Analysis

Table 6 presents the serum biochemical profile results. Compared to the treated and control groups, the G2 group’s total protein, globulin, and albumin levels showed a notable rise (*p* < 0.05). The AST and ALT values in the G2 group were significantly less than those in the other treated and control groups. (*p* < 0.05).

**Table 6:** Effect of Technospore ® on Biochemical Analysis of *Oreochromis niloticus*

Parameters	CNT	G1	G2	G3
Globulin (g/dl)	1.81 ± 0.05 ^c^	1.85 ± 0.01 ^c^	2.14 ± 0.03 ^a^	2.05 ± 0.09 ^ab^
Albumin (g/dl)	1.32 ± 0.02 ^ab^	1.32 ± 0.03 ^ab^	1.35 ± 0.01 ^a^	1.29 ± 0.01 ^b^
Total Protein (g/dl)	3.13 ± 0.03 ^c^	3.18 ± 0.03 ^c^	3.49 ± 0.02 ^a^	3.34 ± 0.09 ^b^
AST (U/l)	27.05 ± 0.57 ^ab^	28.59 ± 1.25 ^a^	26.41 ± 0.23 ^b^	28.01 ± 0.65 ^ab^
ALT (U/l)	29.48 ± 0.49 ^ab^	28.75 ± 0.69 ^bcd^	28.18 ± 0.79 ^d^	29.91 ± 0.09 ^a^

The data represent the mean ± SE. A different superscript for the same numbers indicates notable variations (*p* < 0.05) in each row and non-significant for (*P* > 0.05). CNT control group, G1 = (0.2 mg/kg Technospore®), G2 = (0.4 g/kg Technospore®), G3 = (0.8 g/kg Technospore®), *ALT* alanine aminotransferase, *AST* aspartate aminotransferase

### Immunological Parameters

Regarding immunological measures, Phagocytic activity was markedly elevated in all groups that were supplemented than control (p < 0.05). Lysozyme and phagocytic index were dramatically increased (p < 0.05) in the G2 group as opposed to the control group and all treated groups, as demonstrated in Table 7.

**Table 7:** Effect of Technospore ® on Phagocytic activity, Phagocytic index, and lysozyme of *Oreochromis niloticus*

Parameters	CNT	G1	G2	G3
Phagocytic activity	11.35 ± 0.09 ^c^	13.02 ± 0.05 ^b^	13.25 ± 0.60 ^b^	12.29 ± 0.40 ^b^
Phagocytic index	1.06 ± 0.04 ^c^	1.20 ± 0.02 ^b^	1.31 ± 0.01 ^a^	1.20 ± 0.01 ^b^
lysozyme	12.07 ± 0.62 ^e^	12.42 ± 0.05 ^de^	12.80 ± 0.08 ^a^	12.80 ± 0.01 ^bcd^

The data represent the mean ± SE. A different superscript for the same numbers indicates notable variations in each row (*p* < 0.05)

### Hematological Analysis after Challenge with *A. hydrophila*

RBCs, Hb, PCV, MCV, and MCHC, were considerably higher in all treated and challenged groups with *A. hydrophila* than in the control group with the highest level in G2 (*p* < 0.05), and There were not any notable variations between G2 and G3 as described in (Table 8). Regarding the differential leukocytic count after challenge with *A. hydrophila*, The findings showed that G2 had considerably higher WBCs, Heterophils, and lymphocyte percentages (*p* < 0.05) than control and other supplemented groups. No discernible alterations occurred in monocyte percentage between the supplemented and control groups (*p* > 0.05). The percentages of basophil and eosinophil in all supplemented groups were considerably less than in the control group (*p* < 0.05), as Table 9 illustrates.

**Table 8:** Effect of Technospore® on hematological parameters of *Oreochromis niloticus* challenged with *A. hydrophila*

Parameters	CNT	G1	G2	G3
RBCS (×10^6^/mm^3^)	3.24 ± 0.05 ^d^	3.30 ± 0.02 ^cd^	3.53 ± 0.03 ^a^	3.41 ± 0.00 ^b^
HB (g/100 ml)	9.59 ± 0.01 ^d^	10.12 ± 0.04 ^b^	10.72 ± 0.02 ^a^	10.53 ± 0.04 ^a^
PCV (%)	31.5 ± 0.50 ^de^	32.00 ± 0.00 ^cd^	34.50 ± 0.50 ^a^	33.50 ± 0.50 ^b^
MCV	97.22 ± 0.04 ^a^	96.83 ± 0.73 ^ab^	97.59 ± 0.45 ^a^	98.09 ± 1.32 ^a^
MCH	29.62 ± 0.41 ^b^	30.62 ± 0.11 ^a^	30.34 ± 0.23 ^a^	30.85 ± 0.09 ^a^
MCHC	30.46 ± 0.43 ^c^	31.62 ± 0.12 ^ab^	31.09 ± 0.38 ^b^	31.45 ± 0.33 ^ab^

The data represent the mean ± SE. A different superscript for the same numbers indicates notable variations (*p* < 0.05) in each row and for (*P* > 0.05), not significant. CNT = (control group), G1 = (0.2 g/kg Technospore®), G2 = (0.4 g/kg Technospore®), G3 = (0.8 g/kg Technospore®), *RBCs* Red Blood Cells, *HB* Hemoglobin, *PCV* Packed Cell Volume, *MCV* Mean corpuscular volume, *MCH* Mean corpuscular hemoglobin, *MCHC* Mean corpuscular hemoglobin concentration

**Table 9:** Effect of Technospore ® on the differential leukocytic count of *Oreochromis niloticus* challenged with *A. hydrophila*

Parameters	CNT	G1	G2	G3
WBCs (%)	22.38 ± 2.01^c^	22.67 ± 1.33 ^c^	31.16 ± 0.28 ^a^	26.98 ± 0.47 ^b^
Lymphocyte (%)	65.00 ± 2.00 ^c^	74.00 ± 1.00 ^b^	78.00 ± 2.00 ^a^	74.50 ± 0.50 ^b^
Heterophil (%)	10.00 ± 1.00 ^c^	14.50 ± 1.50 ^a^	14.50 ± 0.50 ^a^	11.50 ± 0.50 ^b^
Monocyte (%)	8.50 ± 0.50 ^c^	9.50 ± 0.50 ^bc^	9.00 ± 1.00 ^bc^	8.50 ± 0.50 ^c^
Basophil (%)	2.50 ± 0.50 ^a^	1.00 ± 0.00 ^b^	1.50 ± 0.50 ^b^	1.00 ± 0.00 ^b^
Eosinophil (%)	3.00 ± 1.00 ^a^	1.00 ± 0.00 ^bc^	1.00 ± 0.00 ^bc^	0.50 ± 0.50 ^c^

The data represent the mean ± SE. A different superscript for the same numbers indicates notable variations (*p* < 0.05) in each row and non-significant for (*P* > 0.05). CNT = (control group), G1 = (0.2 g/kg Technospore®), G2 = (0.4 g/kg Technospore®), G3 = (0.8 g/kg Technospore®), *WBCs* white blood cell count

### Blood Biochemical Analysis after Challenge with *A. hydrophila*

According to data presented in (Table 10), after the challenge with *A. hydrophila*; total protein, albumin, and globulin values in the G2 group exhibited a noticeable increase in comparison to the control group and all of the supplemented groups (*p < 0.05*). AST showed a significant decrease (*p < 0.05*) in the G2 group compared to other supplemented and control groups. ALT showed a significant reduction (*p < 0.05*) in all supplemented groups than the control group. Nevertheless, the supplemented groups had no notable variations (*p* > 0.05).

**Table 10:** Effect of Technospore ® on Biochemical analysis of *Oreochromis niloticus* challenged with *A. hydrophila*

Parameters	CNT	G1	G2	G3
Globulin (g/dl)	1.81 ± 0.01 ^d^	1.87 ± 0.02 ^c^	2.05 ± 0.07 ^a^	1.94 ± 0.07 ^b^
Albumin (g/dl)	1.33 ± 0.01 ^c^	1.31 ± 0.01 ^c^	1.41 ± 0.04 ^a^	1.38 ± 0.01 ^ab^
Total Protein (g/dl)	3.14 ± 0.00 ^d^	3.18 ± 0.01 ^cd^	3.47 ± 0.03 ^a^	3.32 ± 0.08 ^b^
AST (U/l)	38.82 ± 0.18 ^a^	31.09 ± 0.54 ^b^	30.06 ± 0.19 ^c^	30.59 ± 0.42 ^bc^
ALT (U/l)	43.06 ± 2.78 ^a^	33.71 ± 0.43 ^c^	33.50 ± 0.68 ^c^	33.66 ± 0.48 ^c^

The data represent the mean ± SE. A different superscript for the same numbers indicates notable variations (*p* < 0.05) in each row and non-significant for (*P* > 0.05). CNT = (control group), G1 = (0.2 g/kg Technospore®), G2 = (0.4 g/kg Technospore®), G3 = (0.8 g/kg Technospore®), *ALT* alanine aminotransferase, *AST* aspartate aminotransferase

### Immunological Parameters after *A. hydrophila* Challenge

In terms of immunological measurements after challenge with *A. hydrophila*, the G2 group exhibited significantly higher levels of phagocytic activity, phagocytic index, and lysozyme measured against the other treatment groups as well as the control group (*p* < 0.05). as displayed in Table 11.

**Table 11:** Effect of Technospore® on Phagocytic activity, Phagocytic index, and lysozyme of *Oreochromis niloticus* challenged with *A. hydrophila*

Parameters	CNT	G1	G2	G3
Phagocytic activity	11.43 ± 0.05 ^e^	13.28 ± 0.17 ^bc^	13.70 ± 0.64 ^b^	12.52 ± 0.37 ^d^
Phagocytic index	1.10 ± 0.01 ^d^	1.18 ± 0.01 ^c^	1.41 ± 0.01 ^a^	1.26 ± 0.00 ^b^
lysozyme	12.42 ± 0.39 ^c^	12.61 ± 0.07 ^c^	14.59 ± 0.26 ^a^	12.98 ± 0.19 ^bc^

The data represent the mean ± SE. A different superscript for the same numbers indicates significant differences in each row (*p* < 0.05)

### Relative Gene Expression

**Fig. 1:**
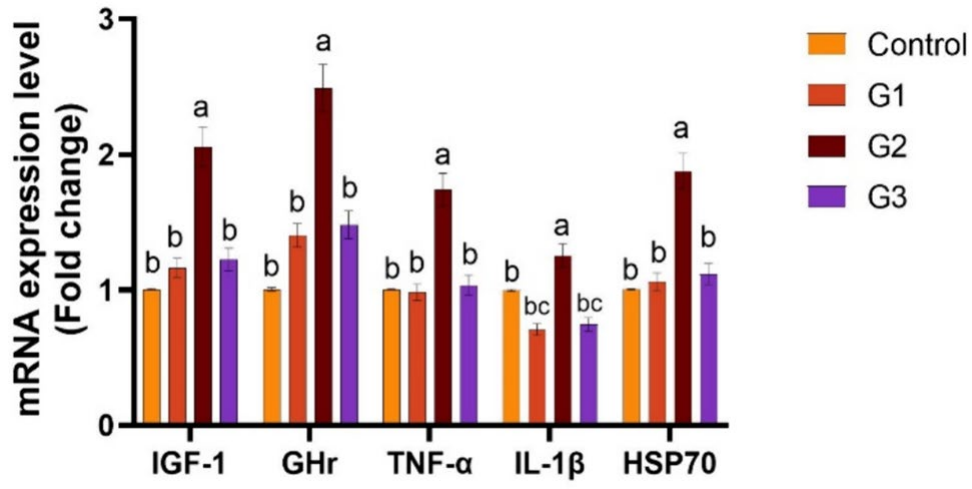
*O. niloticus* liver gene expression (IGF-1, GHR, TNF-α, IL-1β, and HSP70) in different groups fed diets containing varying doses of Technospore®. G1 = (0.2 g/kg Technospore®), G2 = (0.4 g/kg Technospore®), G3 = (0.8 g/kg Technospore®). The presented data represent the mean ± SEM. The values with a different superscript between columns indicate significant differences (*p* < 0.05)

**Fig. 2:**
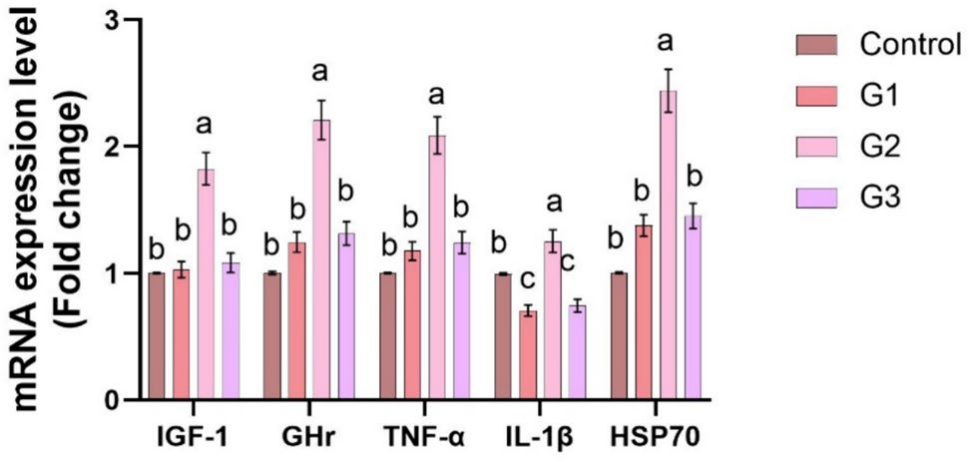
*O. niloticus* liver gene expression (IGF-1, GHR, TNF-α, IL-1β, and HSP70) following the *A. hydrophila* challenge in different groups fed diets containing varying doses of Technospore®. G1 = (0.2 g/kg Technospore®), G2 = (0.4 g/kg Technospore®), G3 = (0.8 g/kg Technospore®). The presented data represent the mean ± SEM. The values with a different superscript between columns indicate significant differences (*p* < 0.05)

**Fig. 3:**
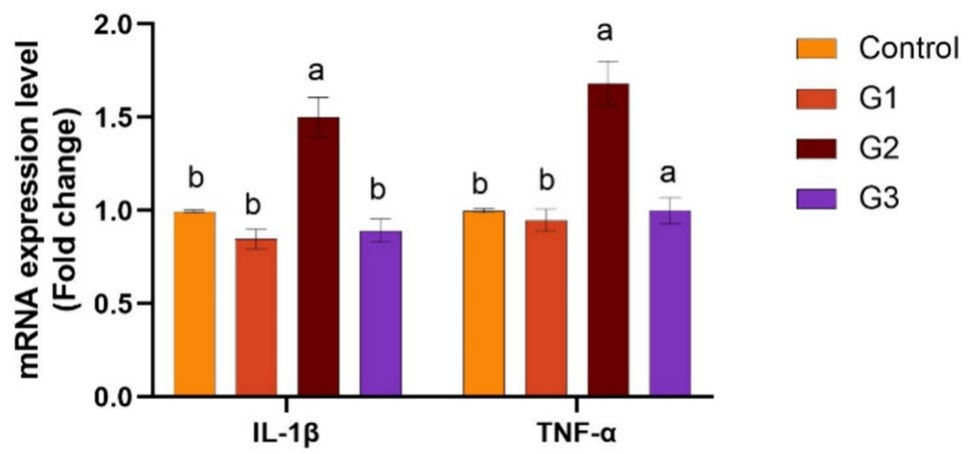
*O. niloticus* spleen gene expression (IL-1β and TNF-α) in different groups fed diets containing varying doses of Technospore®. G1 = (0.2 g/kg Technospore®), G2 = (0.4 g/kg Technospore®), G3 = (0.8 g/kg Technospore®). The presented data represent the mean ± SEM. The values with a different superscript between columns indicate significant differences (*p* < 0.05)

**Fig. 4:**
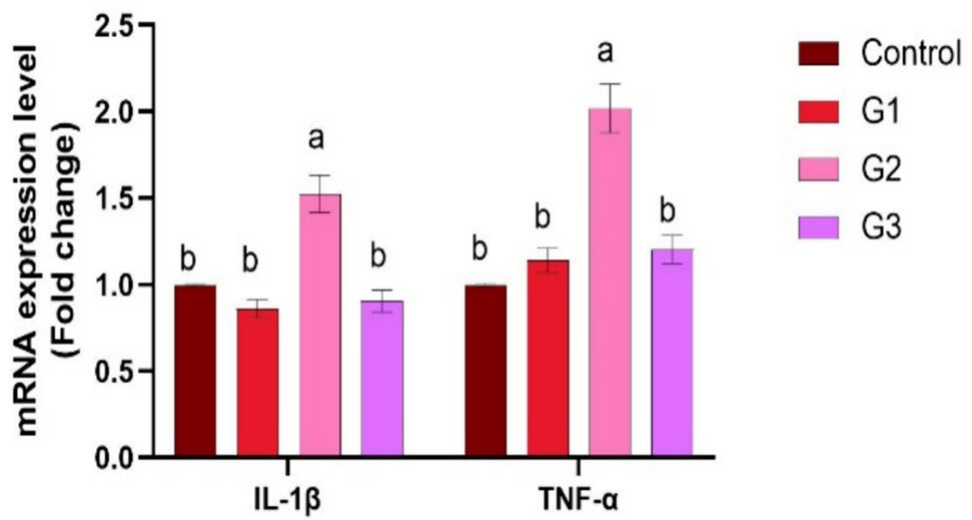
*O. niloticus* spleen gene expression (IL-1β and TNF-α) following the *A. hydrophila* challenge in different groups fed diets containing varying doses of Technospore®. G1 = (0.2 g/kg Technospore®), G2 = (0.4 g/kg Technospore®), G3 = (0.8 g/kg Technospore®). The presented data represent the mean ± SEM. The values with a different superscript between columns indicate significant differences (*p* < 0.05)

**Fig. 5:**
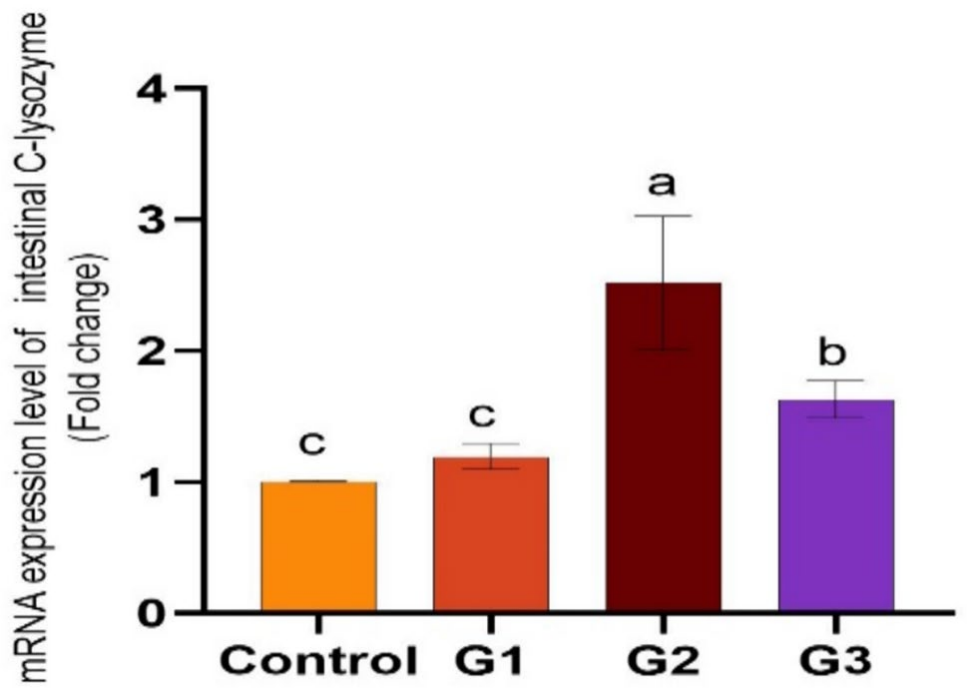
*O. niloticus* Gene expression of intestinal C-lysozyme gene in different groups fed diets containing varying doses of Technospore®. G1 = (0.2 g/kg Technospore®), G2 = (0.4 g/kg Technospore®), G3 = (0.8 g/kg Technospore®). The presented data represent the mean ± SEM. The values with a different superscript between columns indicate significant differences (*p* < 0.05)

**Fig. 6:**
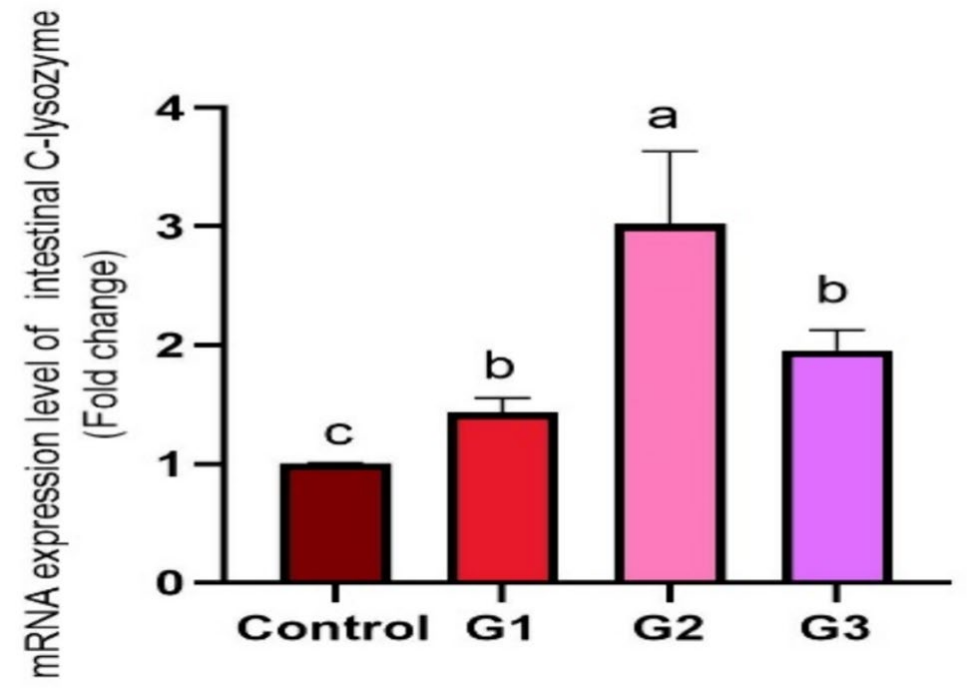
*O. niloticus* Gene expression of intestinal C-lysozyme gene following the *A. hydrophila* challenge in different groups fed diets containing varying doses of Technospore®. G1 = (0.2 g/kg Technospore®), G2 = (0.4 g/kg Technospore®), G3 = (0.8 g/kg Technospore®). The presented data represent the mean ± SEM. The values with a different superscript between columns indicate significant differences (*p* < 0.05)

**Fig. 7:**
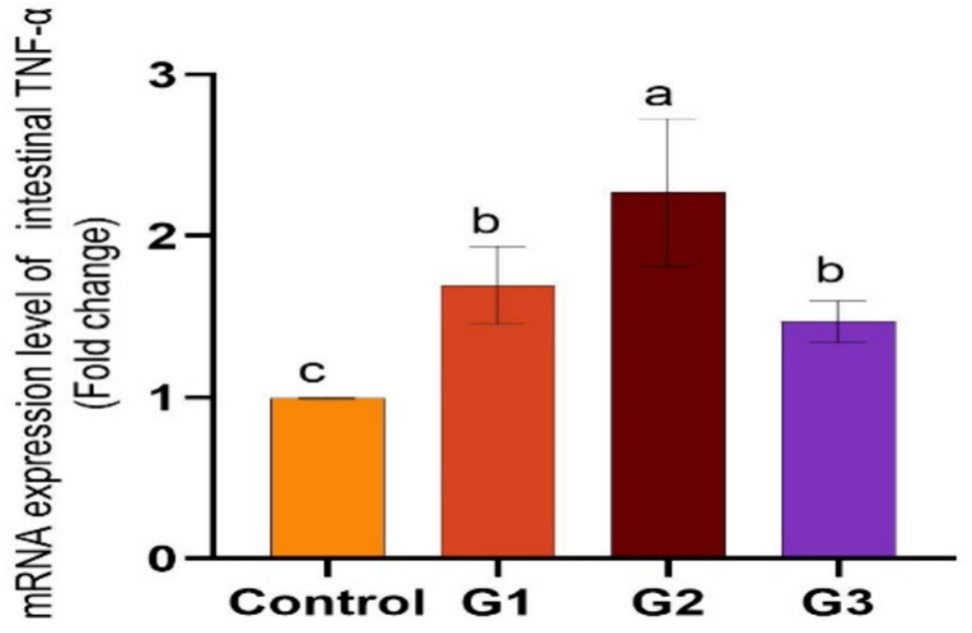
*O. niloticus* Gene expression of intestinal TNF-α gene in different groups fed diets containing varying doses of Technospore®. G1 = (0.2 g/kg Technospore®), G2 = (0.4 g/kg Technospore®), G3 = (0.8 g/kg Technospore®). The presented data represent the mean ± SEM. The values with a different superscript between columns indicate significant differences (*p* < 0.05)

**Fig. 8:**
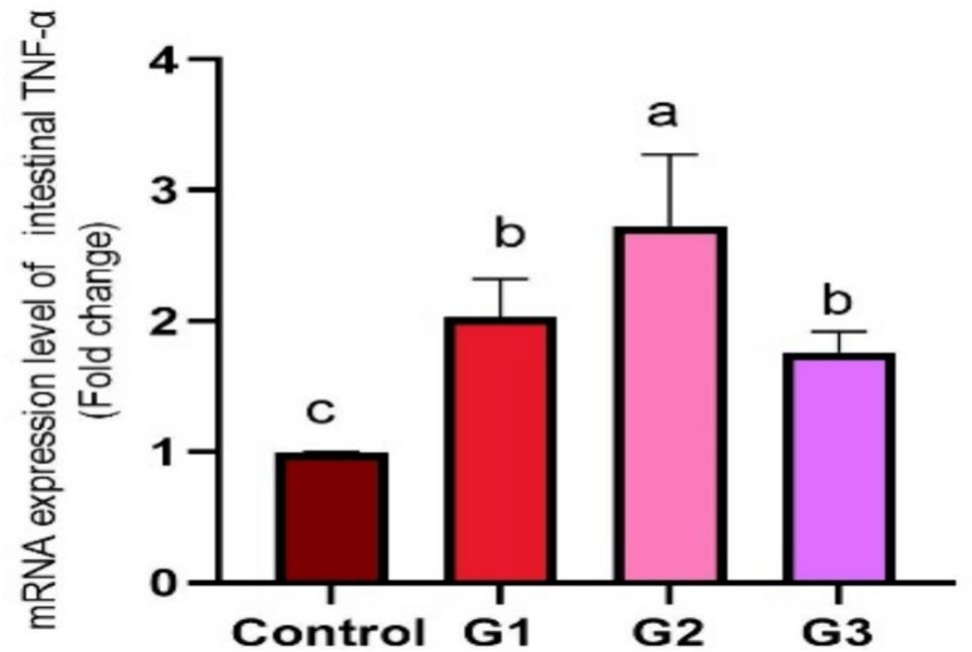
*O. niloticus* Gene expression of intestinal TNF-α gene following the *A. hydrophila* challenge in different groups fed diets containing varying doses of Technospore®. G1 = (0.2 g/kg Technospore®), G2 = (0.4 g/kg Technospore®), G3 = (0.8 g/kg Technospore®). The presented data represent the mean ± SEM. The values with a different superscript between columns indicate significant differences (*p* < 0.05)

Supplying *O. niloticus* with Technospore® raised the liver mRNA transcription of GHR, IGF-1, IL-1β, TNF-α, and HSP70 in G2 (*p* < 0.05) compared to the control and other supplemented groups, Technospore® affecting mRNA expression both before and after the *A. hydrophila* challenge (Figs. 1, 2). Figures 3 and 4 demonstrated that, due to Technospore®'s influence before and after the *A. hydrophila* challenge, the G2-supplemented group had higher spleen IL-1β mRNA expression levels than the control and other supplemented groups. TNF-α mRNA levels in the spleen were also higher in the G2 and G3 groups than in the G1 and control groups (*p* < 0.05). Across all supplemented groups, a substantial rise was observed in the intestinal TNF-α mRNA expression levels and intestinal C-lysozyme genes. In contrast to the control group, the G2 group treated with Technospore® 0.4 g/kg feed displayed a highly significant increase both before and following the challenge with *A. hydrophila* (*p* < 0.05) (Figs. 5, 6, 7 and 8).

### Histopathological Findings

**Fig. 9:**
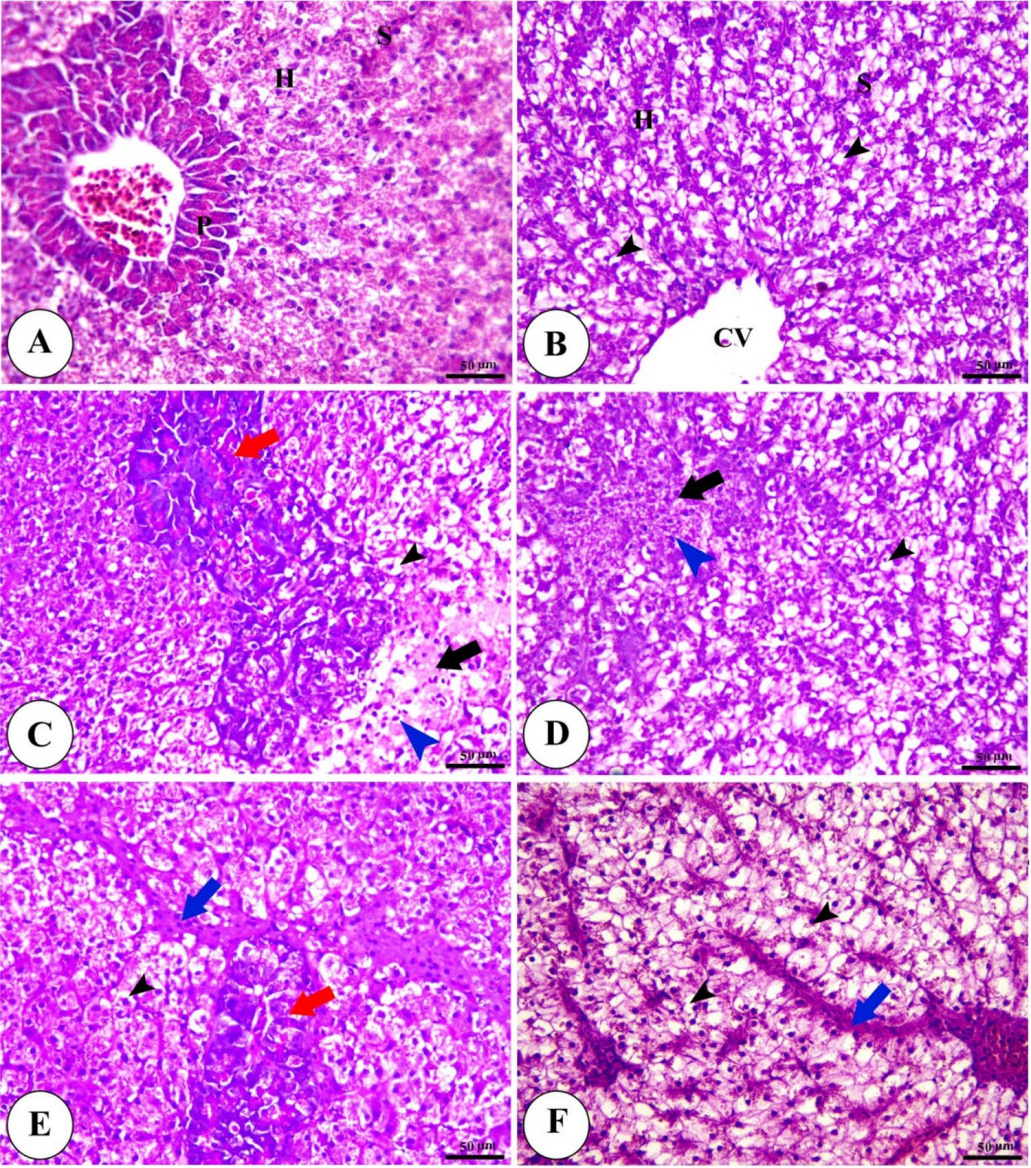
Photomicrograph of H&E stained panel of hepatopancreas of control (**A**), Technospore® 0.8 g/kg (**B**), *A. hydrophila* infected (**C**), Technospore® 0.2 g/kg + *A. hydrophila* (**D**), Technospore® 0.4 g/kg + *A. hydrophila* (**E**) and Technospore® 0.8 g/kg + *A. hydrophila* (**F**), treated groups showing central vein (CV), hepatocytes (**H**), hepatic sinusoids (S), pancreatic acini(P), steatosis of hepatocytes (black arrowheads), focal areas of necrosis (black arrows), inflammatory cells infiltration (blue arrowheads), degeneration of pancreatic cells (red arrows) and congestion of blood vessels (blue arrows)

Hepatopancreas of the control group showed typical morphological features of polyhedral-shaped hepatocytes in the hepatic parenchyma, divided by blood sinusoids and pyramidal cells with basal nuclei and basophilic cytoplasm forming pancreatic acini, while that of Technospore® supplemented groups displayed marked steatosis which increased with the higher concentration of Technospore®. On the other hand, the *A. hydrophila*-infected group shows significant alterations in hepatocyte and pancreatic cell degeneration with focal areas of necrosis and infiltration of inflammatory cells in addition to pancreatic and hepatic vascular congestion. The *A. hydrophila*-infected groups fed on a diet containing Technospore® show a significant decrease in the severity of pathological affection, especially with higher concentrations (0.4 and 0.8 g/kg) of Technospore® as displayed in (Fig. 9).

**Fig. 10:**
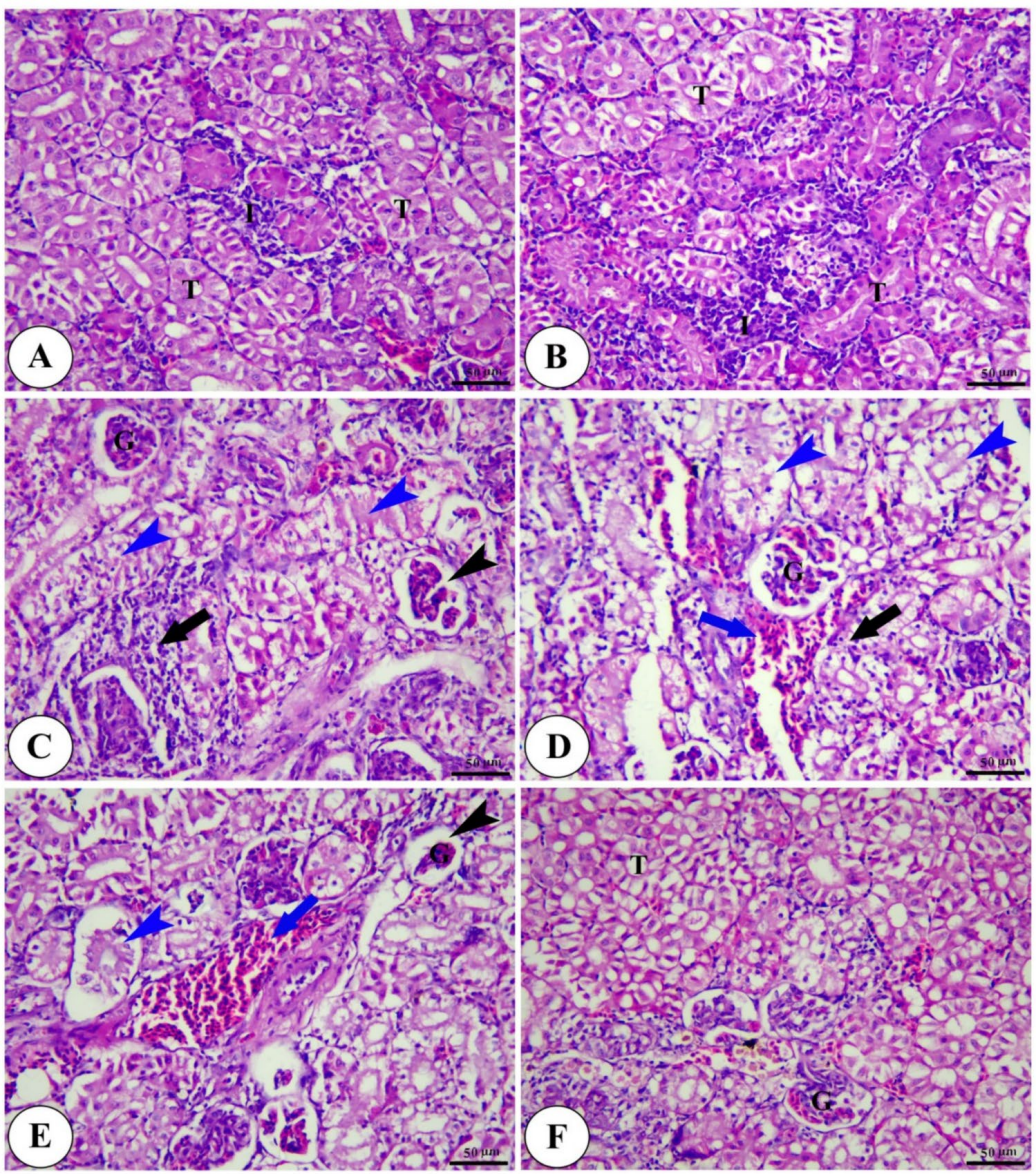
Photomicrograph of H&E stained panel of posterior kidney of control (**A**), Technospore® 0.8 g/kg (**B**), *A. hydrophila* infected (**C**), Technospore® 0.2 g/kg + *A. hydrophila* (**D**), Technospore® 0.4 g/kg + *A. hydrophila* (**E**) and Technospore® 0.8 g/kg + *A. hydrophila* (**F**), treated groups showing renal glomeruli (**G**), renal tubules (T), interstitial tissue (**I**), shrinkage of glomeruli (black arrowheads), degeneration of renal tubules (blue arrowheads), degeneration of interstitial tissue (black arrows) and congestion of blood vessels (blue arrows)

The tail kidney of control and Technospore® groups show typical morphological features of renal parenchyma with renal glomeruli surrounded by Bowman’s capsule, renal tubules, and interstitial hematopoietic and lymphoid tissue. On the other hand, the *A. hydrophila*-infected group shows severe degenerative changes represented by shrinkage of renal glomeruli, severe tubular epithelium atrophy and sloughing, degeneration of interstitial hematopoietic tissue, and congestion of renal blood vessels. The *A. hydrophila*-infected groups fed on a diet containing Technospore® show significant amelioration of renal parenchyma, especially with a higher concentration (0.8 g/kg) of Technospore® as shown in (Fig. 10).

**Fig. 11:**
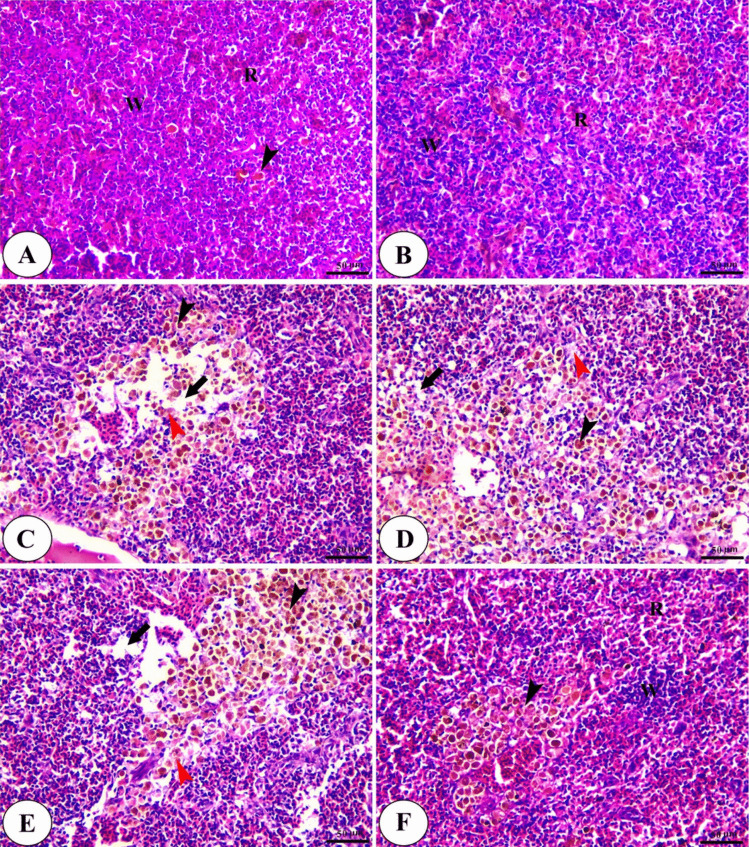
Photomicrograph of H&E stained panel of spleen of control (**A**), Technospore® 0.8 g/kg (**B**), *A. hydrophila* infected (**C**), Technospore® 0.2 g/kg + *A. hydrophila* (**D**), Technospore® 0.4 g/kg + *A. hydrophila* (**E**) and Technospore® 0.8 g/kg + *A. hydrophila* (**F**), treated groups showing red pulp (R), white pulp (W), melanomacrophage center (black arrowheads), degeneration of splenic parenchyma (red arrowheads) and interstitial edema (black arrows)

The spleen of the control and Technospore® groups show the standard histological structure as it is composed of mixed red pulp of an interconnecting network of splenic cords and blood sinusoids in addition to white pulp beside the ellipsoid and a few melanomacrophage centres. The spleen of the *A. hydrophila*-infected group shows severe degenerative changes in splenic parenchyma, interstitial oedema, and focal areas of necrosis. On the contrary, The *A. hydrophila* infected groups fed on a diet containing Technospore® show significant amelioration of splenic parenchyma with increased melanomacrophage centres, especially with higher concentration (0.8 g/kg) of Technospore® as shown in (Fig. 11).

**Fig. 12:**
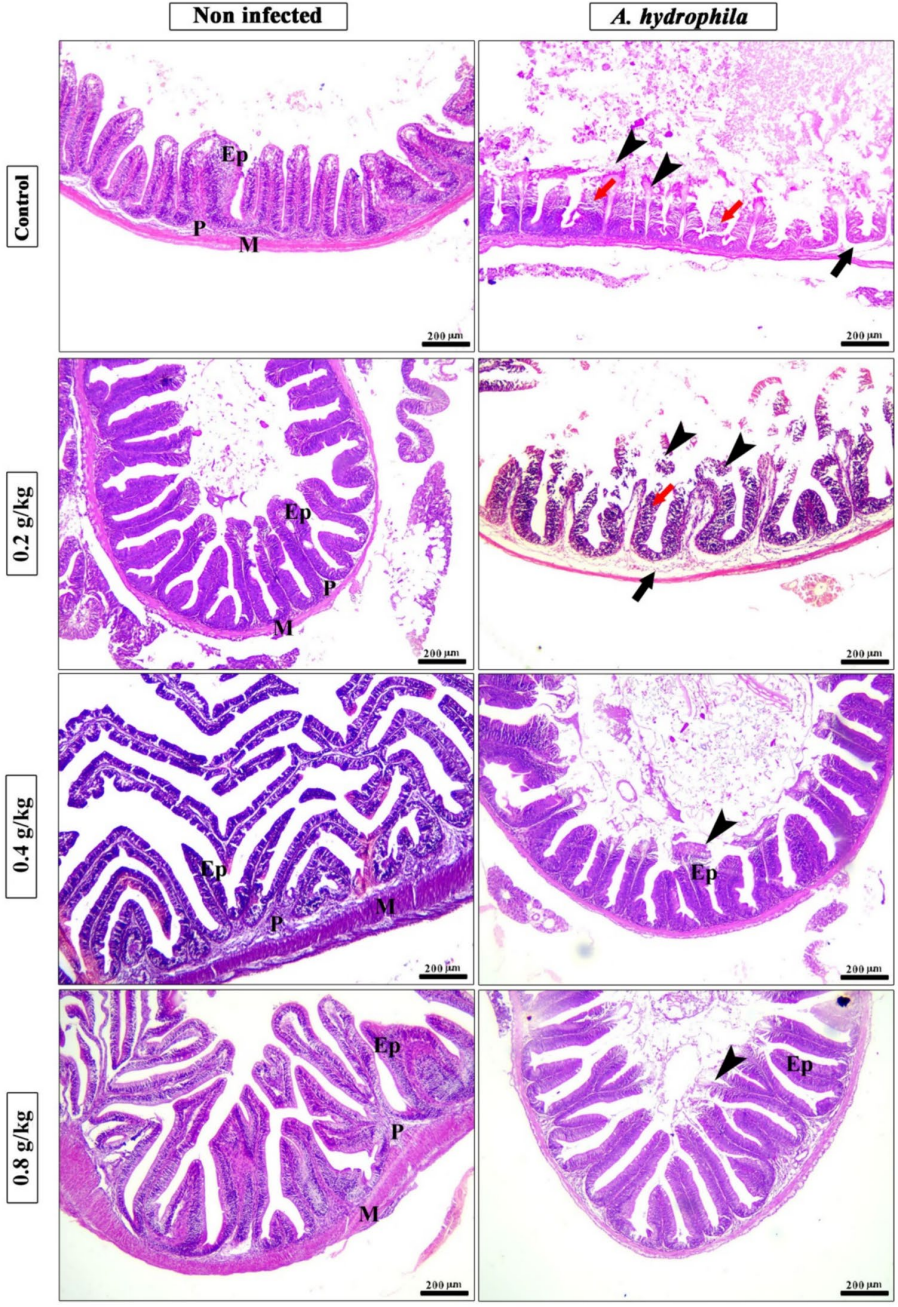
Photomicrograph of H&E stained panel of the anterior part of the intestine of different supplemented groups showing lamina epithelial (Ep), lamina propria (P), lamina muscularis (M), sloughing of the apical part of intestinal villi (black arrowheads), degeneration of lamina epithelial (red arrows) and edema in the lamina propria (black arrows)

Histologically, the intestine’s anterior portion of the control and Technospore® groups is composed of tunica mucosa forming clusters of goblet cells and simple columnar epithelium along intestinal villi and a core of loose connective tissue and lamina muscularis, which is covered externally by tunica serosa. The length and branching of intestinal villi were markedly noticed with the higher concentration of Technospore® (Fig. 12). The morphometric analysis of the intestine revealed a significant increase in villi length, width, crypt depth, villi length/crypt depth, and villi surface area in a dose-dependent pattern especially in medium and high concentration of Technospore® compared with control and low concentration of Technospore® group (Table 12). The results of the histopathology of *A. hydrophila* infected groups displayed severe degenerative changes represented by desquamation and loss of the apical part of intestinal villi beside oedema in the interstitial tissue, especially in control +ve and 0.2 g/kg Technospore® treated groups. The adverse effect was markedly diminished with higher concentrations (0.4 and 0.8 g/kg) of Technospore® (Table 13 and Fig. 12).

**Table 12:** Morphometric analysis of the intestine of *O. niloticus* treated with different concentrations of Technospore®

	Control	G1	G2	G3	P. value
Villi length (μm)	363.0 ± 14.34^b^	395.5 ± 11.79^b^	642.5 ± 30.58^a^	681.6 ± 22.18^a^	< 0.0001
Crypt depth (μm)	63.62 ± 12.62^ab^	44.43 ± 5.01^b^	85.06 ± 6.28^a^	60.68 ± 5.17^ab^	0.0187
villi width (μm)	109.6 ± 3.29^ab^	97.62 ± 6.83^b^	108.9 ± 1.56^ab^	117.9 ± 5.89^a^	0.0658
Villi length/crypt depth	6.56 ± 1.21^b^	9.25 ± 0.81^ab^	7.63 ± 0.31^b^	11.58 ± 1.08^a^	0.0078
Villi surface area (μm^2^)	37,673 ± 955^b^	42,217 ± 782^b^	77,078 ± 2516^a^	84,605 ± 3082^a^	0.0002

CNT=control group, G1 = (0.2 g/kg Technospore®), G2 = (0.4 g/kg Technospore®), G3 = (0.8 g/kg Technospore®). The presented data represent the mean ± SE. A different superscript for the same numbers indicates notable variations (*p* < 0.05) in each row

**Table 13:** Scoring of histopathological lesion of hepatopancreas, kidney, spleen and intestine of *O. niloticus*

Groups lesion	Pre-challenge with *A. hydrophila*				Post-challenge with *A. hydrophila*			
	CNT	G1	G2	G3	CNT	G1	G2	G3
Hepatopancreas								
Steatosis	+	+	++	++	+	+	+	+
Focal necrosis	–	–	–	–	+++	++	+	–
Congestion of blood vessels	–	–	–	–	+++	++	+	+
Inflammatory cells	–	–	–	–	++	++	–	–
Kidney								
Shrinkage of glomeruli	–	–	–	–	+++	++	+	+
Degeneration of tubules	–	–	–	–	+++	++	+	+
Degeneration of interstitial tissue	–	–	–	–	+++	++	+	–
Congestion of blood vessels	–	–	–	–	+++	++	+	–
Spleen								
Degeneration and necrosis	–	–	–	–	+++	++	+	+
Interstitial edema	–	–	–	–	++	++	+	–
Melanomacrophage centers	+	+	+	+	+++	+++	++	+
Intestine								
Degeneration of villi	–	–	–	–	+++	+++	+	+
Interstitial edema	–	–	–	–	++	++	–	–
Branching of villi	–	–	++	+++	–	–	–	+

CNT=Control group, G1 = (0.2 g/kg Technospore®), G2 = (0.4 g/kg Technospore®), G3 = (0.8 g/kg Technospore®), Lesion scoring: (−) none, (+) Mild, (++) Moderate, (+++) Severe

## Discussion

Fish health may be affected by the type and duration of probiotic treatment. It is known that the most efficient method of ensuring the effectiveness of colonizing fish’s digestive system with probiotic microorganisms is to supplement with probiotic foods. Probiotics can be introduced into aquaculture systems as feed or water additives [36, 37].

The primary goals of the aqua feeds sector are to maintain the manageability of fish in the aquaculture system by providing preventative health care through a variety of healthy ways, as well as better development and the resilient response of the vast majority of cultured fish species. The primary factor influencing the immune health of cultivated fish species is their nutritional condition, which ultimately determines a higher degree of assurance [38, 39].

A functional nutritional supplement is a crucial management technique for enhancing farmed fish’s resistance to infectious diseases and boosting general growth metrics. Because antibiotic-resistant bacteria are more likely to emerge in these environments and harm aquaculture, consumers, terrestrial animals, and the environment, The public has expressed alarm over antimicrobial use in aquaculture [40].

The outcomes of our feeding experiment demonstrated that adding Technospore® (*B. coagulans*) to the diets of *O. niloticus* significantly affected the growth performance metrics. *B. coagulans*, present in Technospore®, has been linked to improved feed efficiency and growth of all supplemented fish groups with the highest values in the G2 group; our findings were aligned with [41]. Beneficial bacterial strains that multiply in the gastrointestinal system use epithelial cells to help with food absorption and digestion [42]. Microbes that are beneficial and predominate in the gut microbiome also lessen the detrimental effects of pathogenic microorganisms on gut immunity [43]. Consequently, the entire body’s immune system depends on intestinal immunity and health [44]. According to this theory, *B. coagulans* in Technospore® had impacted *O. niloticus’s* performance.

The potential significance of *B. coagulans* could be associated with their role in improving feed intake; our results showed improved feed intake in all supplemented groups with Technospore®; the results are in agreement with [45, 46], mentioned that *B. coagulans* promote the feed palatability, which in turn results in increased feed efficiency also *Bacillus* sp. was fed to *O. niloticus* to enhance feed efficiency and growth performance.

When fish consume Technospore® (*B. coagulans*), blood biochemical measures are typically employed to detect potential changes in the fish’s overall health status [47]. Concerning the hematological parameters, in this current research, all parameters were increased across the supplemented groups with the highest level in the G2 group pre and post-challenge with *A. hydrophila*; the findings were supported by [48].

Meanwhile, the G2 group experienced a more significant rise in WBCs and heterophil% than control and other supplemented groups before and after the *A. hydrophila* challenge. These elevated levels may be due to the enhanced defense response of Technospore® feed supplementation. The obtained result is similar to those stated previously by [49, 50].

Compared to normal values, it is commonly claimed that fish hematological and biochemical variables raised or decreased are caused mainly by diet additives. [51]. Fish given *B. coagulans* were shown to have higher levels of albumin, globulin, and total protein than the control group, with the G2 group having the highest values both before and after the challenge with *A. hydrophila*. Since it has been exhibited that *Bacillus* species increase the total protein content of Nile tilapia, our results are consistent with [52]. Probiotics, therefore, increase intestine immune responses against infections and increase the total protein quantity in fish meal [53].

Liver enzymes like alanine aminotransferase and aspartate aminotransferase. They act as indicators for the health and function of the liver by regulating the transfer of alpha-amino acid amino group function to alpha-keto acid. Significant amounts of ALT and AST are found in animal blood, and these enzymes are usually produced when liver cells are injured. [54]. In the current investigation, AST and ALT significantly decreased in all Technospore®-supplemented groups, with the G2 group exhibiting the lowest levels before and after the *A. hydrophila* challenge. Our findings concur with those of [55], who discovered that probiotic-supplemented meals considerably reduced the levels of ALT and AST in Nile tilapia when compared to the control group.

The current study investigated that phagocyte activity, phagocyte index, and lysozyme activity. Were fundamentally boosted in all *O. niloticus* supplemented groups pre and post-challenge with *A. hydrophila* with the highest levels in the G2 group. The findings align with earlier studies since white blood cells called phagocytes eliminate dead cells and invasive microorganisms [56]. The cellular-immune system protects the fish body against infections, including white blood cells [57]. Non-specific immunity is a significant component of fish body defense. By lysing the peptidoglycan in the cell walls of bacteria, breaking down the membranes of microbe, and causing the bacterial cell wall’s autolytic enzymes to activate, the tiny cationic protein lysozyme kills or destroys bacteria [58]. The current findings showed that Technospore® supplementation substantially altered the dimensions of lysozyme action in the pre and post-challenged groups. Fish were encouraged to eat diets with 0.4 g/kg Technospore®, which expressed the highest serum lysozyme activity. The immune-stimulating properties of dietary Technospore® may cause increased lysozyme action [39]. The results are comparable to those published in [59, 60], which proved the growth of fish, non-specific immunity, and defense against infection with *A. hydrophila* were all considerably impacted by diets supplemented with *Bacillus coagulans*.

The expression of specific genes linked to stress, immunity, and growth is frequently employed to comprehend the genetic foundation of the mechanism of action when researching the effects of functional feed additives on aquatic species [61]. Cells of fish secrete high heat shock protein 70 in reaction to stress, which improves protein integrity and lowers apoptosis [62]. According to the current findings, HSP70 was elevated in tilapia fish-fed diets enriched with Technospore® and challenged with *A. hydrophila*. This finding is related to the potential role that *B. coagulans* may have in maintaining fish health. It is also commonly recognized that growth hormones (GHr) and (IGF-1) control several critical physiological processes in fish, including growth, metabolism, and mineral homeostasis [63]. Our study’s conclusions demonstrated that *B. coagulans’* effects increased tilapia fish expression levels. The outcomes matched those of other previous investigations [64]. Growth-related gene expression was significantly changed by probiotics, indicating a positive impact of these microorganisms on overall fish metabolic processes [65]. It has been calculated that Genes for TNF-α and interleukin maintain development, distinction, and activation to take place throughout immune and inflammatory reactions [66]. Outcomes of the gene study indicated that groups fed *B. coagulans* had increased levels of interleukin-1 beta (IL-Iβ) and TNF-α. These genes’ activation in response to diets supplemented with *B. coagulans* confirmed the probiotics’ capacity to reduce inflammation by attracting and activating neutrophils in affected areas, improving the response capability of the immune system, and enhancing fish health in general [67]. The results of our investigation demonstrated a substantial increase in the mRNA levels of the intestinal TNF-α and intestinal C-lysozyme genes both pre and post-the *A. hydrophila* challenge as opposed to the control group. These results were in line with other earlier studies [68, 69], which found that in *O. niloticus*-fed diets including *Lactococcus lactose* or *Bacillus subtilis* WB60, TNF-α, and C-lysozyme were elevated in the anterior kidney and mid-gut and promoted the host’s defense mechanisms.

One could explain the enhanced feed efficiency by considering *B. coagulans*’ impact on gastrointestinal morphometry indices [70]. The results demonstrated that feeding Fish *B. coagulans* favored the length and branching of intestinal villi, which was especially evident at higher Technospore® concentrations. In comparison to the control and low concentration Technospore® group, the morphometric analysis of the intestine showed a significant increase in villi length, width, crypt depth, villi length/crypt depth, and villi surface area in a dose-dependent pattern, particularly in medium and high concentrations of Technospore®. These results concurred with [71] and supported the function of *B. coagulans* in enhancing intestinal barrier absorption capacity, allowing for adequate nutrition levels for the biological and metabolic processes of Pacific White Shrimp. Additionally, the function of goblet cells in generating glycoprotein and antibacterial compounds to protect the intestinal walls from harmful pathogens is associated with their increasing quantity [72]. Similarly, [73] showed that adding *Bacillus* spp. to tilapia diets enhanced every gut histomorphometric indicator. Improved intestinal mucosal adherence, improved epithelial barriers, associated pathogen adhesion inhibition, competitive exclusion of harmful microorganisms, antimicrobial molecule synthesis, and innate immune system control are among the main mechanisms of action of probiotics [74].

## Conclusion

The study on feeding Nile tilapia (*O. niloticus*) with Technospore® (*Bacillus coagulans*) supplemented diets concludes with significant benefits in growth performance, gut health, and immune response. Supplementation at varying levels (0.2, 0.4, and 0.8 g/kg) improved growth rates and feed efficiency, with notable immune system enhancement against *Aeromonas hydrophila* infection. *B. coagulans* also positively affected gut morphology and increased gene expression related to growth and immunity. This suggests Technospore® as an effective, natural alternative to antibiotics in aquaculture, enhancing tilapia welfare and productivity while promoting sustainable practices.

## Data Availability

The authors confirm that the data supporting the findings of this work are available within the article. Raw data that supports the findings are available upon reasonable request.

## References

[1] USDA (2022) An overview of the aquaculture industry in Egypt (Report No. EG2022-0003). U.S. Department of Agriculture

[2] Prabu E, Rajagopalsamy CBT, Ahilan B, Jeevagan IJMA, Renuhadevi M (2019) Tilapia – an excellent candidate species for world aquaculture: a review. Annual Research Rev Biol 31(3):1–14

[3] Wang AR, Ran CY, Wang B (2019) Use of probiotics in aquaculture of China–a review of the past decade. Fish Shellfish Immunol 86:734–755 30553887 10.1016/j.fsi.2018.12.026

[4] Dawood MAO, Koshio S, Abdel-Daim MM, Doan HV (2019) Probiotic application for sustainable aquaculture. Rev Aquac 11(3):907–924

[5] Kuebutornye FKA, Abarike ED, Lu Y (2019) A review on the application of *Bacillus* as probiotics in aquaculture. Fish Shellfish Immunol 87:820–828 30779995 10.1016/j.fsi.2019.02.010

[6] Akhter N, Wu B, Memon AM, Mohsin M (2015) Probiotics and prebiotics associated with aquaculture: a review. Fish Shellfish Immunol 45(2):733–741 26044743 10.1016/j.fsi.2015.05.038

[7] Ringø E (2020) Probiotics in shellfish aquaculture. Aquac Fish 5(1):1–27

[8] Yang G, Peng M, Tian X, Dong S (2017) Molecular ecological network analysis reveals the effects of probiotics and florfenicol on intestinal microbiota homeostasis: an example of sea cucumber. Sci Rep 7:1–12. 10.1038/s41598-017-05312-1 28684750 10.1038/s41598-017-05312-1 PMC5500473

[9] Sánchez B, Delgado S, Blanco-Míguez A, Lourenço A, Gueimonde M, Margolles A (2017) Probiotics, gut microbiota, and their influence on host health and disease. Mol Nutr Food Res 61:1600240. 10.1002/mnfr.20160024026 10.1002/mnfr.201600240 27500859

[10] Thomas LV, Suzuki K, Zhao J (2015) Probiotics: a proactive approach to health. A symposium report. Br J Nutr 114:1–15. 10.1017/S0007114515004043 26548336 10.1017/S0007114515004043

[11] Amoah K, Huang QC, Tan BP (2019) Dietary supplementation of probiotic *Bacillus coagulans* ATCC 7050, improves the growth performance, intestinal morphology, microflora, immune response, and disease confrontation of Pacific white shrimp, *Litopenaeus vannamei*. Fish Shellfish Immunol 87:796–808 30790661 10.1016/j.fsi.2019.02.029

[12] Manjula T (2018) Growth promoting potential and colonization ability of probiotics (*Bacillus coagulans* and *Bacillus subtilis*) on the freshwater prawn, *Macrobrachium rosenbergii* post-larvae. Insights Biol Med 2:007–018

[13] Fu RQ, Chen DW, Tian, G. (2019) Effect of dietary supplementation of *Bacillus coagulans* or yeast hydrolysates on growth performance, antioxidant activity, cytokines and intestinal microflora of growing-finishing pigs. Anim Nutr 5(4):366–372 31890913 10.1016/j.aninu.2019.06.003 PMC6920390

[14] Gu SB, Zhao LNW, Y. et al (2015) Potential probiotic attributes of a new strain of *Bacillus coagulans* CGMCC 9951 isolated from healthy piglet feces. World J Microbiol Biotechnol 31(6):851–863 10.1007/s11274-015-1838-x 25752235

[15] Chang XL, Kang MR, Shen YH et al (2021) *Bacillus coagulans* SCC-19 maintains intestinal health in cadmium exposed common carp (*Cyprinus carpio L.*) by strengthening the gut barriers, relieving oxidative stress and modulating the intestinal microflora. Ecotoxicol Environ Saf 228:112977 34781134 10.1016/j.ecoenv.2021.112977

[16] Zhou YH, Zeng ZHX, YB et al (2020) Application of *Bacillus coagulans* in animal husbandry and its underlying mechanisms. Animal 10(3):454 10.3390/ani10030454 PMC7143728 32182789

[17] EFSA Panel on Additives and Products or Substances used in Animal Feed (FEEDAP) et al (2020) Safety and efficacy of TechnoSpore®(Bacillus coagulans DSM 32016) for piglets, other growing Suidae, chickens for fattening, other poultry for fattening and ornamental birds. EFSA J 18:e06158. 10.2903/j.efsa.2020.6158 32874333 10.2903/j.efsa.2020.6158 PMC7448007

[18] Fath El-Bab AF, Majrashi KA, Sheikh HM, Shafi ME, El-Ratel IT, Neamat-Allah ANF, El-Raghi AA, Elazem AYA, Abd-Elghany MF, Abdelnour SA, Abduh MS, Jaremko M, Naiel MAE (2022) Dietary supplementation of Nile tilapia (*Oreochromis niloticus*) with β-glucan and/or *Bacillus coagulans*: synergistic impacts on performance, immune responses, redox status and expression of some related genes. Front Vet Sci 9:1011715. 10.3389/fvets.2022.1011715 36213404 10.3389/fvets.2022.1011715 PMC9537821

[19] NRC (2011) Nutrient requirements of fish and shrimp. Animal Nutrition Series, National Research Council of the National Academies. The National Academies Press, Washington, DC, USA, p 376

[20] Kumaree KK, Akbar A, Anal AK (2015) Bioencapsulation and application of *lactobacillus plantarum* isolated from catfish gut as an antimicrobial agent and additive in fish feed pellets. Ann Microbiol 65:1439–1445. 10.1007/s13213-014-0982-0

[21] Naiel MA, Abdelghany MF, Khames DK, El-hameed A, Samah A, Mansour EM et al (2022) Administration of some probiotic strains in the rearing water enhances the water quality, performance, body chemical analysis, antioxidant and immune responses of Nile tilapia, *Oreochromis niloticus*. Appl Water Sci 12:1–13. 10.1007/s13201-022-01733-0

[22] Stoskopf M (1993) Fish medicine. WB Saunders company Harcourt Brace Jovanovich Inc., Philadelphia, London, Toronto, Montreal, Sydney, Tokyo

[23] Balasubramaniam P, Malathi A (1992) Comparative study of hemoglobin estimated by Drabkin’s and Sahli’s methods. J Postgrad Med 38:8 1512732

[24] Dacie J, Lewis S (1991) Miscellaneous tests. In: Practical Haematology, 7th edn. Churchill Livingstone, London, pp 227–257

[25] Blaxhall P, Daisley K (1973) Routine haematological methods for use with fish blood. J Fish Biol 5:771–778. 10.1111/j.1095-8649.1973.tb04510.x

[26] Wilkinson J, Baron D, Moss D, Walker P (1972) Standardization of clinical enzyme assays: a reference method for aspartate and alanine transaminases. J Clin Pathol 25:940. 10.1136/jcp.25.11.940 4648539 10.1136/jcp.25.11.940 PMC477570

[27] Demers NE, Bayne CJ (1997) The immediate effects of stress on hormones and plasma lysozyme in rainbow trout. Develop Comp Immunol 21:363–373. 10.1016/S0145-305X(97)00009-8 10.1016/s0145-305x(97)00009-8 9303274

[28] Moustafa EM, Dawood MAO, Assar DH, Omar AA, Elbialy ZI, Farrag FA, Shukry M, Zayed MM (2019) Modulatory effects of fenugreek seeds powder on the histopathology, oxidative status, and immune related gene expression in Nile tilapia (*Oreochromis niloticus*) infected with *Aeromonas hydrophila*. Aquac 515:734589

[29] Kawahara E, Ueda T, Nomura S (1991) In vitro phagocytic activity of white spotted shark cells after injection with *Aeromonas salmonicida* extracelluar products. Gyo Ken Japan 26(4):213–214

[30] Omar AA, Moustafa EM, Zayed MM (2016) Identification and characterization of virulence- associated genes from pathogenic *Aeromonas Hydrophila* strains. WVJ 6(4):185–192

[31] Cruickshank R (1975) Medical Microbiology: a guide to diagnosis and control of infection. E and S Livingston Ltd., Edinburgh and London, p 888

[32] Ibrahim MD, Shahed IB, Abo El-Yazeed H, Korani H (2011) Assessment of the susceptibility of poly culture reared African catfish and Nile Tilapia to *Edwardsiella tarda*. J Amer Sci 7(3):779–786

[33] Reed L, Muench H (1938) A simple method of estimating fifty percent end points. American J Trop Med Hyg 27:493–497

[34] Livak KJ, Schmittgen TD (2001) Analysis of relative gene expression data using real-time quantitative PCR and the 2^−11CT^ method. Methods 25:402–408. 10.1006/meth.2001.1262 11846609 10.1006/meth.2001.1262

[35] Bancroft JD, Gamble M (2007) In: Ed T (ed) Theory and practice of histological techniques. Churchill Livingstone, London

[36] Welker TL, Lim C (2011) Use of probiotics in diets of Tilapia. J Aquac Res develop 1:014

[37] Mei GY, Carey CM, Tosh S, Kostrzynska M (2011) Utilization of different types of dietary fibers by potential probiotics. Canad J Microb 57:857–865 21958046 10.1139/w11-077

[38] Kiron V (2012) Fish immune system and its nutritional modulation for preventive health care. Anim Feed Sci Technol 173(1):111–133

[39] Dawood MAO, Koshio S, Abdel-Daim MM, Van Doan H (2018) Probiotic application for sustainable aquaculture. Rev Aquac 11(3):907–924. 10.1111/raq.12272

[40] Bharathi S, Cheryl A, Rajagopalasamy CBT, Uma A, Ahilan B, Aanand S (2019) Functional feed additives used in fish feeds. Int J Fish Aquat Stud 7(3):44–52

[41] Soltani M, Ghosh K, Hoseinifar SH, Kumar V, Lymbery AJ, Roy S (2019) Genus *Bacillus*, promising probiotics in aquaculture: aquatic animal origin, bio-active components, bioremediation and efficacy in fish and shellfish. Rev Fish Sci Aquacult 27:331–379. 10.1080/23308249.2019.1597010

[42] Kavitha M, Raja M, Perumal P (2018) Evaluation of probiotic potential of *Bacillus* spp. isolated from the digestive tract of freshwater fish *Labeo calbasu*. Aquacult Rep 11:59–69. 10.1016/j.aqrep.2018.07.001

[43] Burr G, Gatlin D, Ricke S (2005) Microbial ecology of the gastrointestinal tract of fish and the potential application of prebiotics and probiotics in finfish aquaculture. J World Aquac Soc 36:425–436. 10.1111/j.1749-7345.2005.tb00390.x

[44] Dawood MA (2021) Nutritional immunity of fish intestines: important insights for sustainable aquaculture. Rev Aquac 13:642–663. 10.1111/raq.12492

[45] Wang Y (2011) Use of probiotics *Bacillus coagulans, Rhodopseudomonas palustris* and *Lactobacillus acidophilus* as growth promoters in grass carp (*Ctenopharyngodon idella*) fingerlings. Aquac Nutr 17:372–378. 10.1111/j.1365-2095.2010.00771.x

[46] Ridha MT, Azad IS (2012) Preliminary evaluation of growth performance and immune response of Nile tilapia *Oreochromis niloticus* supplemented with two putative probiotic bacteria. Aquaculture Res 43:843–852. 10.1111/j.1365-2109.2011.02899.x

[47] Dawood MAO, Magouz FI, Salem MFI, Abdel-Daim HA (2019) Modulation of digestive enzyme activity, blood health, oxidative responses and growth-related gene expression in GIFT by heat-killed *Lactobacillus plantarum* (L-137). Aquac 505:127–136

[48] Abd El-Rhman AM, Khattab YA, Shalaby AM (2009) *Micrococcus luteus* and *Pseudomonas* species as probiotics for promoting the growth performance and health of Nile tilapia (*Oreochromis niloticus*). Fish Shellfish Immunol 27:175–180 19361560 10.1016/j.fsi.2009.03.020

[49] Irianto A, Austin B (2002) Probiotics in aquaculture. J Fish Dis 25:633–642

[50] Yongjian X, Yanbo W, Junda L (2014) Use of *Bacillus coagulans* as a dietary probiotic for the common carp, *Cyprinus carpio*. Aquacul Soc 45:403–411

[51] Femi-Oloye OP, Owoloye A, Olatunji-Ojo AM, Abiodun AC, Adewumi B, Ibitoye BO et al (2020) Effects of commonly used food additives on haematological parameters of Wistar rats. Heliyon 6:05221. 10.1016/j.heliyon.2020.e05221 10.1016/j.heliyon.2020.e05221 PMC7569304 33102847

[52] Azevedo RV, Pereira SL, Cardoso LD, Andrade DRD, Vidal Júnior MV (2016) Dietary mannan oligosaccharide and *Bacillus subtilis* in diets for Nile tilapia (*Oreochromis niloticus*). Anim Sci 38:347–353. 10.4025/actascianimsci.v38i4.31360

[53] Hoseinifar SH, Sun YZ, Wang A, Zhou Z (2018) Probiotics as means of diseases control in aquaculture, a review of current knowledge and future perspectives. Front Microbiol 2429. 10.3389/fmicb.2018.02429 10.3389/fmicb.2018.02429 PMC6194580 30369918

[54] Kumar V, Makkar HPS, Becker K (2011) Nutritional, physiological haematological responses in rainbow trout (*Oncorhynchus mykiss*) juvenile fed detoxified jatropha curcas kernel meal. Aquac Nutr 17:415–467

[55] Soltan MA, El-Laithy SMM (2008) Effect of probiotics and some spices as feed additives on the performance and behaviour of Nile tilapia, *Oreochromis niloticus*. Egypt J Aquat Biol Fish 12(2):63–80

[56] Secombes C, Fletcher T (1992) The role of phagocytes in the protective mechanisms of fish. Annu Rev Fish Dis 2:53–71. 10.1016/0959-8030(92)90056-4

[57] Haghighi M, Rohani MS (2013) The effects of powdered ginger (*Zingiber officinale*) on the haematological and immunological parameters of rainbow trout *Oncorhynchus mykiss*. J Med Plant Herbal Therapy Res 1:8–12. 10.33500/jmphtr.2013.1.002

[58] Whang I, Lee Y, Lee SO, Jung M-J, Choi S-J, CY. et al (2011) Characterization and expression analysis of a goose-type lysozyme from the rock bream *Oplegnathus fasciatus*, and antimicrobial activity of its recombinant protein. Fish Shellfish Immunol 30:532–542. 10.1016/j.fsi.2010.11.025 21167286 10.1016/j.fsi.2010.11.025

[59] Lara-Flores M (2011) The use of probiotic in aquaculture: an overview. Int Res J Microbiol 2:471–478

[60] Akhil G, Paromita G, Asha D (2014) Dietary supplementation of probiotics affects growth, immune response and disease resistance of *Cyprinus carpio* fry. J Fish Shellfish Immuno 41:113–119 10.1016/j.fsi.2014.08.023 25160796

[61] Elbahnaswy S, Elshopakey GE (2020) Differential gene expression and immune response of Nile tilapia (*Oreochromis niloticus*) challenged intraperitoneally with *Photobacterium damselae and Aeromonas hydrophila* demonstrating immunosuppression. Aquac 526:735364

[62] Jun Q, Hong Y, Hui W, Didlyn KM, Jie H, Pao X (2015) Physiological responses and HSP70 mRNA expression in GIFT tilapia juveniles, *Oreochromis niloticus* under short-term crowding. Aquac Res 46:335–345. 10.1111/are.12189

[63] Douros JD, Baltzegar DA, Mankiewicz J, Taylor J, Yamaguchi Y, Lerner DT et al (2017) Control of leptin by metabolic state and its regulatory interactions with pituitary growth hormone and hepatic growth hormone receptors and insulin like growth factors in the Tilapia (*Oreochromis mossambicus*). Gen Comp Endocrinol 240:227–237. 10.1016/j.ygcen.2016.07.017 27449341 10.1016/j.ygcen.2016.07.017 PMC6291831

[64] Dawood MAM, El-Sharawy AE-S, Atta ME, Elbialy AM, Abdel- Latif ZI, HM. et al (2020) The role of β-glucan in the growth, intestinal morphometry, and immune-related gene and heat shock protein expressions of Nile tilapia (*Oreochromis niloticus*) under different stocking densities. Aquaculture 523:735205. 10.1016/j.aquaculture.2020.735205

[65] Ibrahem MD (2015) Evolution of probiotics in aquatic world: potential effects, the current status in Egypt and recent prospectives. J Adv Res 6:765–791. 10.1016/j.jare.2013.12.004 26644914 10.1016/j.jare.2013.12.004 PMC4642160

[66] McGeachy MJ, Chen Y, Tato CM, Laurence A, Joyce-Shaikh B, Blumenschein WM et al (2009) The interleukin 23 receptor is essential for the terminal differentiation of interleukin 17-producing effector T helper cells in vivo. Nat Immunol 10:314–324. 10.1038/ni.1698 19182808 10.1038/ni.1698 PMC2945605

[67] Ai Q, Mai K, Zhang L, Tan B, Zhang W, Xu W et al (2007) Effects of dietary β-1, 3 glucan on innate immune response of large yellow croaker, *Pseudosciaena crocea*. Fish Shellfish Immunol 22:394–402. 10.1016/j.fsi.2006.06.011 16928452 10.1016/j.fsi.2006.06.011

[68] Ghanbari M, Kneifel W, Domig KJ (2015) A new view of the fish gut microbiome: advances from next-generation sequencing. Aquaculture 448:464–475

[69] Won S, Hamidoghli A, Choi W, Park Y, Jang WJ, Kong IS, Bai SC (2020) Effects of Bacillus subtilis wb60 and Lactococcus lactis on growth, immune responses, histology and gene expression in Nile tilapia, *Oreochromis niloticus*. Microorganisms 8:68 31906334 10.3390/microorganisms8010067 PMC7023347

[70] Tengjaroenkul B, Smith BJ, Caceci T, Smith SA (2000) Distribution of intestinal enzyme activities along the intestinal tract of cultured Nile tilapia, *Oreochromis niloticus* L. Aquaculture 182:317–327. 10.1016/S0044-8486(99)00270-7

[71] Boonanuntanasarn S, Wongsasak U, Pitaksong T, Chaijamrus S (2016) Effects of dietary supplementation with β-glucan and synbiotics on growth, haemolymphchemistry, and intestinal microbiota and morphology in the Pacific white shrimp. Aquac Nutr 22:837–845. 10.1111/anu.12302

[72] Lauriano E, Pergolizzi S, Aragona M, Montalbano G, Guerrera M, Crupi R et al (2019) Intestinal immunity of dogfish *Scyliorhinus canicula* spiral valve: a histochemical, immunohistochemical and confocal study. Fish Shellfish Immunol 87:490–498. 10.1016/j.fsi.2019.01.049 30711492 10.1016/j.fsi.2019.01.049

[73] Ghalwash HR, Salah AS, El-Nokrashy AM, Abozeid AM, Zaki VH, Mohamed RA (2022) Dietary supplementation with *Bacillus* species improves growth, intestinal histomorphology, innate immunity, antioxidative status and expression of growth and appetite-regulating genes of Nile tilapia fingerlings. Aquac Res 53:1378–1394. 10.1111/are.15671

[74] Bermudez-Brito M, Plaza-Díaz J, Muñoz-Quezada S, Gómez-Llorente C, Gil A (2012) Probiotic mechanisms of action. Ann Nutr Metab 61:160–174. 10.1159/000342079 23037511 10.1159/000342079

